# The New Pharmacopœia

**Published:** 1914-06-27

**Authors:** 


### THE PROFESSION OF MEDICINE.

### The New Pharmacopœia.

WHEN the last edition of the British Pharmacopœia was published in 1898, the medical profession settled down to assimilate the new remedies and combinations of remedies which it contained, and, more important still, the new doses and new strengths of many of the old favourites. The task was made as easy as possible for the doctors by the adoption of as much uniformity in dosages as was practicable, but naturally there was no great enthusiasm displayed over the business of going to school all over again. The authors of text-books, too, had to prepare new editions so as to keep them up to date. Comfort, however, was derived from the reflection that it would be years and years before the new Pharmacopœia would be altered. Vain hope! Within a short time all sorts of deficiencies were discovered in the "new B.P.," and the authors of the "Extra Pharmacopœia," the "Codex," and similar auxiliary compends were busier than ever at their tasks of supplementing the official volume.

Again, the last sixteen years has seen the rise of enormous numbers of synthetic drugs, most of them proprietary and allotted fancy names. Of all these that have any ascertained value in therapeutics the Pharmacopœia must take note, though it deals with them under their proper chemical names, not under their advertised and usually briefer ones. Thus it can readily be seen that the new edition must of necessity vary very considerably from the old one. To give but one further instance, the rise of the physiologically standardised tincture dates entirely from within the present century, and the popularity of the actual alkaloidal active principle of many vegetable drugs rather than of the extract, tincture, or infusion is also a question of the last few years.

This new edition will be the fifth to be published under the authority of the General Medical Council. The first appeared in 1864, the next in 1867, the third in 1885, and the last in 1898. Before 1864 the component sections of the kingdom had each its own Pharmacopœia. In the preparation of the forthcoming volume the General Medical Council and the Pharmaceutical Society have worked in close connection, as they did over the 1898 edition. For some years advisory committes have been dealing with various sub-divisions of the work; in addition, medical and pharmaceutical authorities throughout the Empire have cooperated with suggestions, especially in regard to the adapting of the new Pharmacopœia to the requirements of all parts of the British Dominions.

From time to time the Committee of Reference in Pharmacy has published reports and recommendations about various matters in connection with the British Pharmacopœia. Not only have the researches of very many independent workers been considered most thoroughly, but in some cases special researches to elucidate particular points have been put in hand. The two principal editors of the new edition are Professor Nestor Tirard, of King's College Hospital, and Professor Greenish, of the Pharmaceutical Society of Great Britain. But their task is so gigantic that the number of those on whom they have relied for help must be very great.

A good many rumours, more or less definite, are in circulation concerning changes, omissions, and additions which may be looked for in the new edition. It may be that many of these are simply intelligent anticipations, based upon the current trend in medical and pharmaceutical developments. At any rate, it is declared that these rumours are wholly unauthorised, and that they can only have been derived from an unjustifiable use of a "highly confidential" copy of the uncompleted proof of the text. This proof has not yet undergone its final revision by the Pharmacopœia Committee, and has therefore not been submitted to the General Medical Council for approval and adoption. Official warning is given that some of the statements that are being made are inaccurate; and it is, of course, obvious that if they happen to be accurate at the time of leaking out, eleventh-hour alterations of the proofs might make them quite inaccurate before publication takes place.

To the medical profession any new edition of the Pharmacopœia causes somewhat mixed sensations. The advantages of being provided with official standards for all sorts of drugs which did not exist sixteen years ago, of the elimination of useless preparations, and of the inclusion of more advantageous ones are largely counterbalanced by the expense and bother of a new text-book, by the trouble entailed in mastering its contents and in committing sections of it to memory, and by that upsetting of old-established habits and customs which most men detest profoundly. But the new British Pharmacopœia is at least indispensable, whatever other adjective one applies to it; so the only thing to do, when it appears (it is promised for September), is to get a copy and study it with a cheerful spirit, inwardly invoking Providence not to render necessary any more new editions in one's life-time, or at least for five-and-twenty years.

